# Development and validation of a machine learning model to detect psychiatric symptoms in Huntington’s disease using speech analysis

**DOI:** 10.1371/journal.pone.0350118

**Published:** 2026-07-01

**Authors:** Quang Tuan Rémy Nguyen, Hadrien Titeux, Rachid Riad, Renaud Massart, Graca Morgado, Katia Youssov, Emmanuel Dupoux, Anne-Catherine Bachoud-Lévi

**Affiliations:** 1 Département d’Études Cognitives, École normale supérieure, PSL University, Paris, France; 2 Université Paris-Est Créteil, Faculté de médecine, Créteil, France; 3 Inserm U955, Institut Mondor de Recherche Biomédicale, Équipe E01 NeuroPsychologie Interventionnelle, Créteil, France; 4 AP-HP, Centre de référence Maladie de Huntington, Service de Neurologie, Hôpital Henri Mondor-Albert Chenevier, Créteil, France; 5 NeurATRIS, Mondor Node, Créteil, France; 6 CNRS 8554, Laboratoire de Sciences Cognitives et Psycholinguistique, PSL University, Paris, France; 7 École des hautes études en sciences sociales EHESS, Paris, France; 8 Inserm, Centre d’Investigation Clinique 1430, AP-HP, Hôpital Henri Mondor, Créteil, France; University of Montreal: Universite de Montreal, CANADA

## Abstract

Huntington’s disease (HD) causes progressive disability through motor, psychiatric, and cognitive symptoms. Machine learning speech analysis can detect motor and cognitive symptoms of HD, but not yet psychiatric symptoms. This study investigated whether speech analyses can detect the presence of psychiatric symptoms in HD. Audio recordings of six narrative tasks (cookie-theft picture description, red-riding hood storytelling, most recent 24 hours recalling, happy, sad, or angry storytelling) were prospectively collected from subsequent genetically confirmed HD participants from the BIOHD and REPAIR CAPIT-HD-Beta cohorts at the Hospital Henri-Mondor, Créteil. Speech therapists blindly annotated speech samples to allow extraction of three types of features: linguistic, LASER, and acoustic features. Psychiatric symptoms in participants were detected using the Problem Behaviors Assessment Short version (PBA-s). Machine learning classifier models were trained on 80% of the 89 participants before being tested on the remaining 20% of individuals. F1-scores were calculated and compared to chance. Linguistic features detected obsessive/compulsive behavior (OCB) with all but joy task, and best with the cookie task (F1-score: 0.67, confidence interval [0.47–0.86] (p ≦ 0.001)). They also best detected depression with the red-riding hood (F1 score 0.66, [0.45–0.87], p ≦ 0.001), apathy with the joy task (0.60, [0.39–0.81], p ≦ 0.001), but not irritability. LASER features best detected OCB (0.65, [0.45–0.84], p ≦ 0.001), depression (0.60, [0.40, 0.80], p ≦ 0.01) and apathy (0.61, [0.37, 0.86], p ≦ 0.001) from the red-riding hood task, but not irritability. Acoustic features best detected depression (0.63, [0.46, 0.80], p ≦ 0.001) and OCB (0.60, [0.43, 0.77], p ≦ 0.001) but not apathy nor irritability. This study showed that speech analyses can detect obsessive/compulsive behaviors, depression, and apathy in HD participants but not irritability. Linguistic and LASER features provided the most consistent detections, but acoustic features also detected depression and OCB, highlighting their complementary role for psychiatric characterization in HD.

## Introduction

Huntington’s disease (HD) is an autosomal dominant neurodegenerative disorder, related to the mutation of the Huntingtin (HTT) gene on chromosome 4. It progresses towards disability through a triad of motor, psychiatric, and cognitive symptoms. Among those, psychiatric manifestations encompassing anxiety, despair, obsessive-compulsive behaviors, apathy and hallucinations, affect up to 98% of patients [1]. They can be the first manifestation of the disease [2], with major consequences like social withdrawal or suicide [1]. They constitute the major factor of burden in HD at moderate stages [3]. However, despite their high weight on patients and caregivers’ relationships and quality of life, psychiatric disorders are difficult to assess; physicians might rather focus on motor symptoms, easier to detect and assess [4]. In addition, psychiatric disorders not only rarely constitute the endpoint of therapeutic trials and also may constitute an exclusion criterion for being part of studies [4]. Currently, psychiatric symptoms in HD are assessed in a yearly face-to-face interview conducted by trained specialists using the Problem Behaviors Assessment Short version (PBA-s) [5]. This 11-item semi-structured interview captures depression, suicidal ideation, anxiety, irritability, lack of initiative, obsessive/compulsive behavior, and hallucinations [1,5]. Although valuable, this approach has its limitations: neurologists and psychiatrists trained in this rare disease are not so common, patients may come unaccompanied and not express their symptoms in the absence of a caregiver or even avoid consultations because of psychiatric disorders [6]. Thus, more accessible and objective tools for monitoring psychiatric status are mandatory in HD.

Speech analysis appears as a readily accessible and affordable marker due to its ease of recording. The recent rise of machine learning methods, capable of analyzing complex and high-dimensional data, has paved the way for the development of speech-based biomarkers. Speech encompasses two main components: (1) acoustic features representing articulation including prosody derived through signal processing and (2) linguistic features representing all aspects of spoken language production.

To date, there has been an overwhelming prevalence of acoustic analyses in literature in neurodegenerative disorders. This is explained by relative ease of automatic extraction, compared to linguistic features which remains resource-intensive to annotate, especially when articulation is impaired [7]. Indeed, acoustic analyses are effective for psychiatric disorders (mostly depression) and neurologic disorders (mostly Parkinson’s (PD) or Alzheimer’s diseases (AD)) [8–15], including HD. They allow distinguishing manifest HD from controls [16], from prodromal stages [17], or predicting disease severity for motor, functional, and cognitive scores [18–20].

Nevertheless, prior work suggests that linguistic features (though grammatical, lexical or syntactical patterns) might be essential in some cases, such as diagnosing psychotic disorders [10], AD [13], or mild cognitive impairment [13], or depression in AD [21] and PD [22]. In HD, Gallezot et al [23] compared acoustic and linguistic performance (using language-agnostic embeddings, Language-Agnostic SEntence Representations, LASER) for emotional expression (not psychiatric symptoms), showing that patients struggle to express emotions and that linguistic analyses may be more effective in discriminating emotions than acoustic one. To our knowledge, speech-based approaches to PBA-S–assessed symptoms in HD has not yet been investigated. This calls for a comprehensive approach using both acoustic and linguistic feature (including classical linguistic analysis and LASER). The acoustic features consisted of the Geneva Minimalistic Acoustic Parameter Set (GeMAPS) created from an international initiative for emotion and psychiatric analyses [9,23,24]. Since no equivalent consensus exists in linguistic features, an original combination described in literature combining lexical, syntactical, emotional or grammatical characteristics [9–14,21] was constructed, along LASER [23].

Here, we developed and validated a machine learning procedure to analyze speech extracted from narrative tasks to detect the psychiatric symptoms assessed by the PBA-s, in participants carrying the HTT mutation.

## Materials and methods

### Participants

Gene carriers of the HTT gene were included in the National Reference Center for Huntington’s disease at the Hospital Henri-Mondor Créteil, France, in two prospective longitudinal cohorts assessing new biomarkers and tools in HD: BIOHD (NCT01412125) and CAPIT-HD beta from Repair-HD (NCT03119246). The inclusion criteria were: (1) HD genetically confirmed with at least 38 CAG repeats on the mutant HTT gene of HD (2) Age ≧ 18 years old, and (3) availability of a speech records (which were run since 07/06/2018). The exclusion criteria were cognitive inability to perform the tasks, the presence of a neurological or psychiatric disorder unrelated to HD, and incomplete speech assessments. Participants were considered premanifest (pre-HD) if their Total Motor Score (TMS) was less than 5 and their Total Functional Capacity (TFC) [25] equaled 13 [26].

### Standard protocol approvals, registrations, and patient consents

All participants signed an informed written consent. Ethical approval was given by the institutional review board from Henri Mondor Hospital (Créteil, France, 2003−09) for BIOHD study and St Louis Hospital (Paris, France, 2016−06) for CAPIT-HD beta. It complied with the Helsinki Declaration, current Good Clinical Practice guidelines, and local laws and regulations.

### Core assessment

Certified examiners evaluated participants’ motor, functional, and cognitive capabilities using different scores including all parts of the Unified Huntington’s Disease Rating Scale (UHDRS) [26]. The functional decline was assessed using the TFC: scores from 11–13 represent Stage I (autonomous participants); 7–10, Stage II; 3–6, Stage III; 1–2, Stage IV; and score of 0 Stage V (bedridden patients) [25]. The disease burden score, that gives an estimate of an individual’s lifetime exposure to mutant huntingtin age × [CAG – 35.5] [27], was calculated. The composite UHDRS (cUHDRS) as formulated in [28] was calculated. The HD Integrated Staging System (HD-ISS) [29] was calculated when applicable (≧ 40 CAG repeats): HD-ISS 0–1 indicates participants carrying the mutation with no pathological alterations (0) or at most striatal atrophy (1), HD-ISS 2, the appearance of motor (TMS) or cognitive (Symbol Digit Modalities Test) symptoms, and HD-ISS 3 the onset of functional decline (with TFC and independence scale).

The psychiatric and behavioral disorders were annually assessed with the PBA-s [1,5] by certified neurologists. The severity (0 = absent, 1 = doubtful, 2 = mild, 3 = moderate, and 4 = severe) and frequency (0 = never/almost never, 1 = rarely, 2 = occasionally, 3 = frequently, and 4 = daily/almost daily) of each neuropsychiatric symptom were independently scored and then multiplied to provide the subscore for each symptom. The subscores were then aggregated into five scores of symptoms: depression (depressed mood, suicidal mood, and anxiety subscores), irritability (irritability and aggressivity), apathy (apathy), psychosis (delusion and hallucination), and obsessive/compulsive behavior (OCB, perseverative and obsessive-compulsive) [5,30].

### Speech assessments

To balance feasibility and ecological validity, our protocol included six narrative tasks alternating neutral and emotional content, designed to elicit sufficient material without exhausting participants.

Neuropsychologists proposed during interviews the six narrative speech tasks at the end of the core assessment. The participants were asked to tell, in the following order, their most recent 24 hours (“24h”), a sad story (“sadness”), the red-riding hood story (“red-riding hood”), an angry story (“anger”), the description of the cookie-theft picture (“cookie”), and finally a joyful story (“joy”) to avoid ending the experiment with a negative emotion. Neutral tasks (cookie theft, storytelling) were selected based on prior evidence of their utility [12,21], while avoiding overly personal stories that could help identifying the participant. Emotional tasks targeted three universal basic emotions and were included because emotional reactivity can be reliably elicited and contrasted across different affective stimuli [31–33]. All participants completed the tasks in less than 15 minutes.

Speech was recorded on microphones (Zoom H4nPro recorders, sampled at 44.1 kHz with a 16-bit resolution). Using the software Praat [34] and Seshat platform [35], speech therapists (blindly of clinical assessment) provided (1) annotations of the language content in stretches of continuous speech delimited by voice inflections and breathing pauses, and (2) eventual linguistic anomalies when detected. This process took between five to eight hours per interview and was carried out since 07/06/2018 until 21/05/2024.

### Features

To detect psychiatric symptoms, three feature sets were automatically generated. (1) “linguistic” using a linguistics-based approach combining lexical, syntactical, emotional or grammatical characteristics [9–14,21]; (2) “LASER” relying on an artificial neural network approach [36] (previously used in [23]), and (3) “acoustic” based on the consensual set GeMAPS [9,23,24]. A complete description of the features can be found in S1 file. “linguistic” (1) and “LASER” (2) were generated from the annotated stretches of language content, while “acoustic” (3) was generated from participant audio file.

#### Linguistic features set.

A set of 151 linguistic features was identified from literature [9–14,21]. They were quantified by their absolute number of occurrences on the whole task (and when specified with a mean, standard deviation, minimum or maximum value by task or stretch). They were split into five groups:

- Grammatical constituents at the word level: spaCy’s (‘fr core news lg’) [37] automatically identified grammatical constituents (so called part-of-speech or PoS): e.g., Adverb, Pronoun, Verb, Determiner, Coordinating Conjunction, Subordinating Conjunction, Auxiliary, Adposition, Adjective, and Noun). Each spaCy’s PoS was counted as well as its ratio on the whole number of various spaCy’s PoS (count/total of PoS). The number of nouns was also differently counted alone or preceded by a determiner [21]. Pronouns were classified as first, second or third person, singular or plural, disjunctive (e.g., “me” or “them”) or not (e.g., “I” or “they”).
- Syntactic complexity at the stretch level: this included word counts, words per seconds, the number of short stretches (<3 words), total speech time and total silence time. Using spaCy, the dependency tree distances [38] (number of intervening words between two syntactically related words) and links (the number of syntactic relations between words) were calculated. The repetition of word occurrence or of stretch occurrence (by cosine similarity between the stretches) was also assessed.
- Lexical richness: this included word frequency using wordfreq [39] on python and various lexical diversity indices: the Honore statistics, Brunet index, Type of Token Ratio (TTR), and the Moving-average type token ratio (MATTR) [12]. These features counted each token only once, and used both the lexeme (various phonological word forms, e.g., drive, drove, driven) and their lemma (their semantic/syntactic form, e.g., drive) for each word, which allows to infer the morphological inflexion.
- Sentiment analysis: a dedicated French lexicon [40] associated emotional values (positivity, joy, fear, sadness, angry, surprise and disgust) to each word. These features included the sum of the word emotional values for each emotion in each task, and the total number of emotional words. Negations were automatically detected using spaCy to avoid inverting the meaning of the sentence (e.g., “I am not happy” not being a positive value).
- Non-intended productions: Speech therapists annotated various anomalies such as fillers, pauses, interruptions, phonological distortions, semantic errors, morphological errors, neologisms, repetitions, abnormal prosody, omitted, stuttered, phonetized, and unintelligible words during annotation. S1 Fig contains examples of such non intended productions.

#### LASER features set.

LASER, a language-agnostic semantic space model, captures semantic characteristics of words or entire utterances for a variety of languages including French taking text as input [36]. In such a model, semantically similar utterances are represented closely in a Euclidean space. Each stretch was individually embedded into the 1024 LASER vectors and means, and standard deviations were calculated from the entire embeddings. Then, the whole stretches were embedded on the 1024 laser vectors, thus obtaining altogether 3072-dimensional semantic space (mean, SD, and whole).

#### Acoustic feature set.

All features used in the GeMAPS [24], created from an international initiative to provide a common baseline for research in emotion and mental state recognition and publicly available through the openSMILE toolkit [24], were automatically extracted.

### Machine learning procedure

The whole pipeline was implemented using sklearn [41] and designed to balance robustness (given the small sample size relatively to the high dimensionality of the feature sets), simplicity and interpretability (given the originality of combining both component of speech).

**Fig 1:**
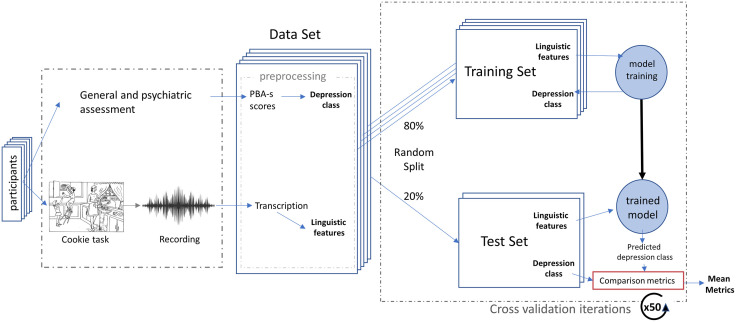
Example of machine learning procedure for the detection of depression symptoms with linguistic features extracted from the cookie task. This procedure was applied for each of the four psychiatric symptom classes (depression, irritability, apathy, and obsessive/compulsive behavior), with each of the three sets of features, extracted from each of the six speech tasks.

A general illustration of the pipeline with application in the depression symptoms detection using the linguistic features from the cookie speech task as an example in Fig 1.

This study used sklearn [41] in python to analyze the features obtained from each of the six speech tasks for each participant. To calculate robust average metrics, 50-fold cross-validation (from steps 1–5) [42] was used. (1) To limit model’s true performance biases, random splits were used: the model was trained on dataset issued from 80% participants (training set) and evaluated it on the data from the 20% remaining participants (test set) [42] (type 2a from the Tripod Statement [43]) (2) Standardization of features was performed using Robust Scaler, thus limiting their intrinsic variability within participants. (3) A logistic regression classifier was trained for detecting psychiatric symptoms. To avoid overfitting and improve model generalization, a L2 penalty was used with low regularization coefficient C = 0.01. (4) The psychiatric symptoms were detected for each participant on the test set. The predicted classifications were then compared to the observed psychiatric classification defined by the PBA using several metrics.

The chance level was calculated with a “dummy model” [41] whose prediction reflected distribution of psychiatric symptoms in the training set.

### Metrics and statistical analysis

#### Psychiatric classes.

Participants were classified as symptomatic for a given psychiatric symptom (depression, irritability, apathy, and OCB) if their score on the corresponding assessment reached the cohort median score for that symptom. Psychosis was excluded from the machine learning procedure because it was only observed in a single patient.

#### Main metric and secondary metrics.

The primary endpoint was the F1-score that compares directly predicted and observed psychiatric class by combining precision (True positive/ (True positive + False positive)) and sensitivity (Se, True positive/ (true positive + false negative)) per class. When dealing with unbalanced classes, the F1-score mitigates the potential bias towards the majority class [9].


$$\begin{array}{c} F1-score=\frac{2*Sensitivity*Recall}{Sensitivity+Precision} \end{array}$$

Accuracy, sensitivity, specificity (Sp), positive predictive value (PPV), and negative predictive value (NPV) were also reported, as these are indicative of clinical utility. For simplicity, the mean values of these metrics over 50-folds for cross-validation were systematically referred to in the following text.

#### Statistical analysis.

Whether the F1-score differed from the chance level was evaluated; the closer the F1-score is to 1, the better the prediction performance. An independent t-test was used when the normality requirement was met with the Shapiro-Wilk test; otherwise, a non-parametric equivalent (Mann-Whitney U-test). P-values were corrected for multiple comparisons per feature set (linguistic, LASER or acoustic features) and per task (cookie, 24h, red-riding hood, sadness, joy, and anger), thus multiplied by 18.

#### Exploratory post-hoc analysis.

A line plot (S2 Fig) displayed the resulting F1-scores and their confidence interval. The validity of the sample size was assessed by running the machine learning procedure while increasing the size of the training set, resulting in line plots (S3 Fig). A hierarchically-clustered Heatmap (with linguistic features in S4 Fig and acoustic features in S5 Fig) displayed logistic regression coefficients of the features.

## Results

### Population description

Among the selected 99 participants, 10 were excluded for not having completed all the speech tasks, leaving 89 participants included in the machine learning procedure. PBA’ scores were as follows: Irritability mean: 0.71 (standard deviation: 1.5, range: [0.0–8.0]), OCB 1.55 (2.96, [0.0–16.0]), Apathy 0.8, (2.34, [0.0–16.0]), and Depression 5.08 (6.11, [0.0–24.0]). The figure S2 Fig illustrated the score distributions. The median score was four for depression and zero for all the other psychiatric scores: thus, the classification thresholds were set to four for depression and one for the other symptoms.

The excluded participants did not clinically differ from the 89 analyzed (Table 1) with a mean TFC of 11.0 (2.79, [4–13]), cUHDRS 10.65 (4.97, [2.20–17.40]), TMS 23.8 (20.47, [0–55]); four were classified as depressed, three irritable, three obsessed, and one apathetic.

**Table 1:** Demographics and characteristics of the participants. The psychiatric (aggregated) scores and (isolated symptoms) subscores are calculated by multiplying severity by frequency [1,5]. Values are mean (standard deviation, range) or n (number of participants (%)). TFC: Total Functional Capacity. cUHDRS: composite Unified Huntington’s Disease Rating Scale. OCB: Obsessive/compulsive behavior. Study level: 12 = high school degree. N CAG: Number of CAG repeats. ISS: Integrated Staging System. Not applicable if CAG < 40. ^a^Subscore on non-aggregated symptom.

HD-ISS		0-1	2	3	Not applicable
**n**		19	20	47	3
**Sex (M,F)**		(6, 13)	(14, 6)	(18, 29)	(2, 1)
**TFC (mean, sd, range)**		12.89 (0.46, [11.0, 13.0])	12.65 (0.59, [11.0, 13.0])	9.85 (1.92, [5.0, 12.0])	11.67 (2.31, [9.0, 13.0])
**Pre_HD (n)**		19	4	0	2
**Age (mean,sd, range)**		49.11 (4.4, [40.29, 55.92])	52.96 (13.2, [25.39, 72.22])	53.56 (9.98, [34.56, 74.1])	68.49 (17.18, [57.23, 88.26])
**Educational level (number of years)**		14.47 (2.32, [11.0, 18.0])	13.6 (3.47, [9.0, 20.0])	12.79 (2.84, [9.0, 18.0])	13.33 (4.04, [9.0, 17.0])
**CAG triplets**		41.53 (1.43, [40.0, 44.0])	43.2 (2.46, [41.0, 49.0])	43.77 (2.67, [40.0, 54.0])	38.67 (0.58, [38.0, 39.0])
**disease course (mean,sd, range)**		7.63 (4.5, [0.7, 16.3])	6.64 (4.92, [0.3, 15.8])	5.66 (5.08, [0.2, 22.6])	5.63 (5.55, [1.8, 12.0])
**cUHDRS**		17.59 (0.98, [15.68, 18.88])	12.95 (2.8, [8.98, 17.56])	8.6 (2.93, [2.48, 16.09])	14.05 (5.15, [8.57, 18.78])
**Total Motor Score**		0.21 (0.54, [0.0, 2.0])	19.35 (13.71, [0.0, 42.0])	35.4 (14.14, [0.0, 60.0])	10.67 (17.62, [0.0, 31.0])
**Symbol Digit Modalities Test**		56.89 (9.19, [41.0, 78.0])	34.6 (12.08, [17.0, 61.0])	24.39 (9.1, [3.0, 45.0])	40.67 (21.36, [25.0, 65.0])
**Disease burden score**		293.34 (60.63, [185.98, 402.9])	381.87 (58.89, [249.7, 482.43])	423.63 (78.96, [200.75, 655.46])	220.63 (83.44, [143.075, 308.91])
**Depression**	**n**	7 (36.84%)	7 (35.0%)	36 (76.6%)	1 (33.33%)
	**Aggregated score** ^**a**^	4.26 (6.38, [0.0, 19.0])	3.2 (4.92, [0.0, 19.0])	6.21 (6.09, [0.0, 24.0])	1.33 (2.31, [0.0, 4.0])
	**depressed mood**	1.68 (3.4, [0.0, 12.0])	1.0 (2.45, [0.0, 9.0])	1.49 (3.06, [0.0, 12.0])	0.0 (0.0, [0.0, 0.0])
	**suicidal ideation**	0.47 (1.17, [0.0, 4.0])	0.3 (1.34, [0.0, 6.0])	0.06 (0.32, [0.0, 2.0])	0.0 (0.0, [0.0, 0.0])
	**anxiety**	2.11 (2.9, [0.0, 9.0])	1.9 (2.27, [0.0, 8.0])	4.66 (3.51, [0.0, 16.0])	1.33 (2.31, [0.0, 4.0])
**Irritability**	**n**	5 (26.32%)	7 (35.0%)	13 (27.66%)	0 (0.0%)
	**Aggregated score** ^**a**^	0.68 (1.6, [0.0, 6.0])	0.85 (1.46, [0.0, 4.0])	0.7 (1.6, [0.0, 8.0])	0.0 (0.0, [0.0, 0.0])
	**irritability**	0.53 (1.12, [0.0, 4.0])	0.85 (1.46, [0.0, 4.0])	0.6 (1.26, [0.0, 6.0])	0.0 (0.0, [0.0, 0.0])
	**anger**	0.16 (0.69, [0.0, 3.0])	0.0 (0.0, [0.0, 0.0])	0.11 (0.6, [0.0, 4.0])	0.0 (0.0, [0.0, 0.0])
**OCB**	**n**	0 (0.0%)	2 (10.0%)	30 (63.83%)	1 (33.33%)
	**Aggregated score** ^**a**^	0.0 (0.0, [0.0, 0.0])	0.3 (0.98, [0.0, 4.0])	2.79 (3.75, [0.0, 16.0])	1.33 (2.31, [0.0, 4.0])
	**perseveration**	0.0 (0.0, [0.0, 0.0])	0.1 (0.45, [0.0, 2.0])	2.15 (2.87, [0.0, 16.0])	1.33 (2.31, [0.0, 4.0])
	**obsessive-compulsive**	0.0 (0.0, [0.0, 0.0])	0.2 (0.89, [0.0, 4.0])	0.64 (2.63, [0.0, 16.0])	0.0 (0.0, [0.0, 0.0])
**Psychosis**	**n**	0 (0.0%)	0 (0.0%)	1 (2.13%)	0 (0.0%)
	**score**	0.0 (0.0, [0.0, 0.0])	0.0 (0.0, [0.0, 0.0])	0.06 (0.44, [0.0, 3.0])	0.0 (0.0, [0.0, 0.0])
**Apathy**	**n**	1 (5.26%)	0 (0.0%)	19 (40.43%)	0 (0.0%)
	**score**	0.21 (0.92, [0.0, 4.0])	0.0 (0.0, [0.0, 0.0])	1.43 (3.06, [0.0, 16.0])	0.0 (0.0, [0.0, 0.0])

### Speech tasks description

On average, participants completed all six speech tasks in 6 minutes and 39 seconds (standard deviation: 4 minutes and 13 seconds). The mean duration of speech tasks was 66.53 (15.72) seconds, with 188.13 by mean (102.89) words. The red-riding hood story was the longest task, with a mean duration of 92.09 (40.84) seconds and 259.58 (140.36) words, and the cookie task the shortest (mean 44.18 (23.46) seconds, with 124.17 (71.21) words).

### Psychiatric symptoms detection

**Fig 2:**
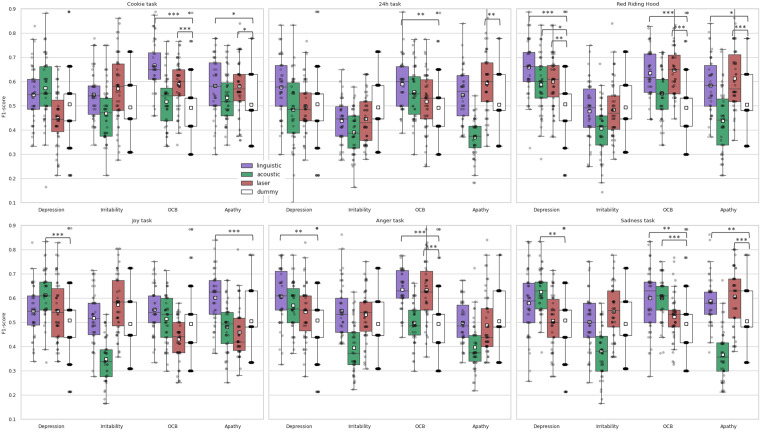
Box plot illustrating the results using both linguistic and LASER sets of features compared to chance level. The boxes extend to the first and third quartiles of the F1-scores across the 50 folds of cross validation. The horizontal line showed the median, and the whiskers show the remainder of the distribution, except for outliers’ points. The square figured the mean within its 95% confidence interval. *: 0.01 < p ≤ 0.05, **: 0.001 < p ≤ 0.01, ***: p ≤ 0.001, these statistics are Bonferroni corrected for multiple comparison.

Linguistic features allowed to detect better than chance the obsessive/compulsive behavior (OCB) with all tasks (cookie, anger and red-riding hood tasks p ≦ 0.001, 24h and sadness p ≦ 0.01) but not with joy task, depression with both the red-riding hood (p ≦ 0.001) and the anger p ≦ 0.01 tasks, apathy with the joy (p ≦ 0.001), sadness (p ≦ 0.01), cookie (p = 0.018) and the red-riding hood (p = 0.012) tasks. LASER features extracted enabled obsessive/compulsive behavior detection from the cookie, red-riding hood and anger tasks (p ≦ 0.001), depression with red-riding hood task (p ≦ 0.01), apathy with red-riding hood, sadness (p ≦ 0.001), 24h (p ≦ 0.01) and cookie (p = 0.020) tasks. Acoustic features enabled obsessive/compulsive behavior detection from the sadness task (p ≦ 0.001), depression with joy, sadness (p ≦ 0.001) and red-riding hood tasks (p = 0.031). Apathy was not detected using acoustic features Irritability was not detected with any of the feature sets. Results are summarized in Table 2, Fig 2 and detailed in S1 Table.

**Table 2:** Results of the statistical analyses between the F1-score from logistic regression of linguistic or LASER features, compared to chance level. The mean value of F1-score was calculated over the 50 folds of cross validation. The table summarizes only the main results with *: 0.01 < p ≤ 0.05, **: 0.001 < p ≤ 0.01, ***: p ≤ 0.001. These statistics are Bonferroni corrected for multiple comparisons. CI95%: 95% Confidence interval. N.S.: non-significant.

		Depression		Irritability		Obsessive/compulsive behavior		Apathy	
Features	Task	Mean	CI95%	Mean	CI95%	Mean	CI95%	Mean	CI95%
Linguistic	Cookie	N.S. N.S.		N.S. N.S.		0.67 ***	[0.47,0.86]	0.58 *	[0.35,0.82]
	24h	N.S. N.S.		N.S. N.S.		0.59 **	[0.40,0.78]	N.S. N.S.	
	Red-riding	0.66 ***	[0.45,0.87]	N.S. N.S.		0.63 ***	[0.44,0.83]	0.58 *	[0.36,0.80]
	Joy	N.S. N.S.		N.S. N.S.		N.S. N.S.		0.60 ***	[0.39,0.81]
	Anger	0.61 **	[0.39,0.82]	N.S. N.S.		0.63 ***	[0.48,0.79]	N.S. N.S.	
	Sadness	N.S. N.S.		N.S. N.S.		0.60 **	[0.35,0.85]	0.59 **	[0.41,0.77]
Acoustic	Cookie	N.S. N.S.		N.S. N.S.		N.S. N.S.		N.S. N.S.	
	24h	N.S. N.S.		N.S. N.S.		N.S. N.S.		N.S. N.S.	
	Red-riding	0.59 *	[0.37,0.81]	N.S. N.S.		N.S. N.S.		N.S. N.S.	
	Joy	0.61 ***	[0.43,0.80]	N.S. N.S.		N.S. N.S.		N.S. N.S.	
	Anger	N.S. N.S.		N.S. N.S.		N.S. N.S.		N.S. N.S.	
	Sadness	0.63 ***	[0.46,0.79]	N.S. N.S.		0.60 ***	[0.43,0.77]	N.S. N.S.	
Laser	Cookie	N.S. N.S.		N.S. N.S.		0.59 ***	[0.40,0.78]	0.58 *	[0.36,0.80]
	24h	N.S. N.S.		N.S. N.S.		N.S. N.S.		0.59 **	[0.34,0.84]
	Red-riding	0.60 **	[0.40,0.80]	N.S. N.S.		0.65 ***	[0.45,0.84]	0.61 ***	[0.37,0.86]
	Joy	N.S. N.S.		N.S. N.S.		N.S. N.S.		N.S. N.S.	
	Anger	N.S. N.S.		N.S. N.S.		0.63 ***	[0.39,0.87]	N.S. N.S.	
	Sadness	N.S. N.S.		N.S. N.S.		N.S. N.S.		0.61 ***	[0.38,0.83]

### Post-hoc analyses

With smaller training sets, results remained consistent with the findings described above (S3 Fig): detection could be achieved with at least 60% of the training set (i.e., 53 participants), even 30% of the training set (i.e., 27 participants) depending of the feature set and speech task. Among linguistic features (S4 Fig), lower syntactic indices, word use and more silences indicated depression, OCB or apathy. Some patterns of pronoun use may also be influential while no clear sentiment analysis pattern could be identified. Among acoustic features influence (S5 Fig), variability of harmonics, loudness peak, formant amplitude or first coefficient of Mel-Frequency Cepstral Coefficient (MFCC) may have a differential role between OCB and depression detection, while lower pitch seemed to indicate both depression and OCB.

## Discussion

This prospective cross-sectional study examined whether speech-based machine learning analyses could detect psychiatric symptoms, as measured by the PBA-s, in 89 French HTT gene carriers from both Bio-HD and Repair HD cohorts. Samples were derived from both emotional (sad, angry, and happy stories) and non-emotional speech (the cookie theft description, the red-riding hood, and the last 24 hours). The machine learning model using linguistic, LASER and acoustic feature sets demonstrated the ability to detect several psychiatric symptoms. None of the models successfully detected irritability, likely reflecting the low irritability levels observed in this cohort (median score: 0,71). Linguistic feature analyses showed the strongest predictive performance, enabling the detection of obsessive-compulsive behavior (OCB), apathy, and depression. LASER features detected OCB and apathy across most speech tasks and detected depression with the red-riding hood task. Acoustic features detected depression in the sadness, joy and red-riding hood tasks, and OCB with sadness task, but did not detect apathy. Overall, these findings extended previous HD speech research, which has primarily focused mainly on motor, cognitive, and functional domains with acoustic analysis, and highlighted the added value of linguistic approaches for the characterization of psychiatric symptoms.

The linguistic features improved the model’s capacity to detect psychiatry in HD, as in previous model targeting emotional expression [23]. This added value of linguistic features was unexpected due to the overwhelming predominance of acoustic-based studies in both psychiatry and neurology [8–10,12,14,15,21,22,44]. The linguistic set comprised grammatical, lexical, syntactic, and affective markers, all of which have been shown to be powerful predictors of psychiatric symptoms, for example reduced lexical diversity, altered syntactic complexity, or the use of affective vocabulary [10,12,21]. Post-hoc analyses further illustrated the complex interactions between these features. Although no single linguistic pattern emerged to detect psychiatric class in this analysis, several linguistic features previously reported may reflect HD symptoms. For example, the use of first‑ and third‑person pronouns have been linked to depression, extended silence duration to apathy, and reduced lexical and syntactic complexity to different psychiatric conditions [9,10,12]. In contrast, LASER embeddings (which primarily encode sentenced-level semantics) performed relatively less consistently than the linguistic feature set. While useful for cross‑linguistic applications, LASER may not capture subtle linguistic mechanisms relevant to psychiatric states. In HD, difficulties in emotional expression or recognition [23,42] may further limit the discriminative power of purely semantic representations. Moreover, LASER features’ high dimensionality (>3,000) could have exceeded the simple logistic regression capabilities.

In contrast, compared to linguistic and LASER feature sets, GeMAPS acoustic features broadly less informative, except for depression, and did not detect apathy. This partial, and relative weaker success of acoustic features may stem from the early presence of dysarthria in HD or medication which may distort acoustic markers [46]. In HD, dysarthria induces imprecise vowel articulation, altered pitch, slower articulation rate, fewer pauses, and prolonged pause ratios, while the antipsychotics medication can cause excessive pitch and loudness variations [46]. These effects may confound the acoustic patterns usually associated with psychiatric disorders (such as reduced pitch variability, energy, fundamental frequency, or speech rate in depression [9,10,44]; this pattern seemed partly found in our post-hoc analysis) while probably leaving linguistic choices relatively intact. Thus, while acoustic features enhanced the performance (particularly for depression detection) linguistic features appeared more robust overall in HD, especially in the context of early confounding effects of dysarthria and treatment on speech production. More broadly, our findings suggest that different feature sets offer complementary strengths, which may enhance the robustness and clinical relevance of speech‑based tools for psychiatric symptom assessment.

Speech‑based studies addressing OCB are scarce in psychiatric literature review [9,10]. Two previous studies [47,48] have examined psychiatric obsessive-compulsive disorders detection: one using speech graph analysis and one using acoustic features in non‑adults populations. In the present study, OCB could be detected with linguistic features, including LASER embeddings, and to a lesser extent acoustic features. A recent review [10] also highlighted the lack of speech‑based studies focusing specifically on apathy and irritability in neurological and psychiatric disorders. Since LASER embeddings have previously been shown to classify emotions in HD [23], the findings suggest a counterintuitively non-straightforward relationship between emotional expression and PBA-s-assessed symptoms. For example, one might expect the “anger” story to be associated with the irritability symptoms. However, irritability levels were low in our cohort: it is possible that participants experienced mild irritability rather than overt anger. Supporting this interpretation, a systematic review [45] showed individuals with HD exhibit reduced recognition of negative vocal emotions (particularly anger), and two recent studies [49,50] showed that HD patient struggle to recall and narrate anger-shared stories and that they express that emotion less.

In the present work, speech analyses did not enable the detection of all psychiatric symptoms across all speech tasks. This could be explained by two interrelated factors: the relatively small sample size, and the mild severity and imbalance of psychiatric symptoms. Here, the sample size was comparable to those reported in the psychiatric speech-based studies (median size fewer than 100 participants [8,9,22,51]). Pérez-Toro et al [22] successfully classified depression in PD with 60 participants. Given the practical constraints of conducting speech-based (even more with linguistics) studies, recommended sample sizes range from 74 to 353 participants per group [9,51], depending on the studied disorders. Notably, our post-hoc analysis confirmed psychiatric symptom detection using even smaller training sets, consistent with previous studies [47,48] that reported OCB classification with fewer than 50 participants.

In addition, the low and unbalanced psychiatric symptoms may have further limited detection performance. Classification thresholds were set at 4 for depression and 1 for other symptoms. Irritability, which could not be detected by our model, scored the lowest scores (median 0 [interquartile range: 0–1]). These values were below those reported in the international HD cohort used to validate PBA-s (median 1 (IQR 0–5) [1] and were consistent with other HD cohorts [1,5]. Similar low values patterns have been reported in PD or AD studies [21,22,52], in which depression scores were also slight or mild at most: unlike dedicated psychiatric studies, neurological cohorts often avoid severe psychiatric symptoms. Future studies including participants with more severe psychiatric symptoms may improve generalizability.

Literature reviews report a wide range of speech tasks used across disorders and feature types [8,9,12]. Spontaneous speech tasks, which aims at capturing ecological language use (word choice, and pauses could be important), are particularly suitable for linguistic analysis than constrained tasks such as reading (more control on sound production or evoked emotions) or sustained vowels (more suitable for some acoustic features) [9]. Spontaneous speech has also been used in acoustic analyses in AD, depression or bipolar disorders [8] and this choice of task aligns with its predominance in literature [8,13,21,22,47,48]. Emotional speech tasks were included with the expectation that they might amplify symptom‑related differences [31–33]. However, aside from detecting depression through acoustic features in the sadness and joy tasks, there was no clear evidence of such an effect in either the linguistic or LASER feature sets, nor did these tasks support the detection of other psychiatric symptoms (particularly irritability). These findings suggest that neutral tasks may suffice for extracting linguistic markers, though further work is needed to clarify the role of emotional tasks and HD‑specific peculiarities.

The finding that linguistic features play an important role in psychiatric symptom detection in HD raises two main considerations related to their inherently language-dependent nature. First, our dataset was collected at Henri Mondor hospital in French and the pipeline, although based on a simple logistic regression classifier, will require language-specific training to ensure its generalizability to other languages. However, the nature of dysarthria and the role of medication, which limit the direct use of Automatic Speech Recognition (ASR), are likely to affect speech similarly across languages, making it plausible that their impact on acoustic features would not differ substantially between languages. Although LASER was trained on 200 languages, its modest performance in this study suggests that future work should explore alternative language‑agnostic embeddings to facilitate cross‑linguistic transfer. Second, the present study relied on time-consuming manual annotations. The use of ASR could reduce this burden in future work; however, dedicated evaluations remain necessary, as ASR accuracy declines in individuals with neurological disorders and varies across algorithms [7]. Future studies should therefore compare gold‑standard human annotations with ASR‑derived features to assess the predictive value of linguistic features in HD and potentially other neurological diseases, across multiple languages.

## Conclusions

This study provides the first classification of PBA-s assessed psychiatric symptoms in carriers of the mutant HTT gene using speech-based analysis. The findings demonstrate the potential of speech to detect psychiatric symptoms, including obsessive-compulsive behavior, in individuals with premanifest and early-stage Huntington’s Disease. Linguistic features appear particularly relevant for psychiatric symptom detection in HD, potentially due to the influence of dysarthria on acoustic parameters. Although complete automation of the linguistic features extraction remains challenging, this study highlighted the importance of combining linguistic and acoustic speech features in the development of future psychiatric evaluation tools in HD.

## Supporting information

S1 Fig Example of speech production in cookie task, after manual transcription (actual words) and annotation of non-intended productions by speech pathologist. The second part is a translation in English that tried to keep the French errors. non intended productions (order of first apparition in the French example): ‘&= ‘: non linguistic additions, ‘&-’: filler, ‘↫’: stuttered word, ‘( )’: omitted word, ‘ [:] [*p]’: phonological distortion, ‘<> [//]’ revision (i.e., when participant change (revise) the intended sentence), ‘<> [x2]”: repetition, ‘ [:] [*p]’: morphological error, ‘ [_]’: abnormal prosody.
(TIF)

S1 Table Complete results of the models using each of the three different feature sets with each of the six different speech tasks, detecting each of the psychiatric symptoms. The last row was Chance level. Abbreviations: DEP depression, IRR irritability, OCB obsessive compulsive behavior, APA apathy, se sensibility, sp specificity, ppv positive predictive value, npv negative predictive value, p_val p_value, NS non-significant.
(DOCX)

S2 Fig Class counts for each psychiatric symptom. Grey bars indicate participants with PBA‑s scores below the median (classified as negative for the symptom), while red bars indicate participants with PBA‑s scores above the median (classified as positive for the symptom).
(TIF)

S3 Fig Evolution of F1-score (y-axis) when the model is trained with increasing subsets of training set (%, x-axis). *: 0.01 < p ≤ 0.05, **: 0.001 < p ≤ 0.01, ***: p ≤ 0.001, these statistics were calculated only with the complete training set (same statistic than Fig 2 and Table 2) and are Bonferroni corrected for multiple comparison.
(TIF)

S4 Fig Hierarchically clustered heatmap of linguistic features, in conditions when results were above chance level. Logistic regression coefficients were converted to odds ratios and subsequently clustered within the sub‑categories of the linguistic feature sets to facilitate readability. Abbreviations: DEP depression, OCB obsessive compulsive behavior, APA apathy.
(TIF)

S5 Fig Hierarchically clustered heatmap of acoustic GeMAPS features, in conditions when results were above chance level. Logistic regression coefficients were converted to odds ratios and subsequently clustered to facilitate readability. Abbreviations: DEP depression, OCB obsessive compulsive behavior.
(TIF)

S1 File Complete description of the three feature sets. (DOCX)
