# Supplementary figures and images for: Development and validation of a machine learning model to detect psychiatric symptoms in Huntington’s disease using speech analysis
Source: PLoS One. 2026 Jul 1;21(7):e0350118. doi: 10.1371/journal.pone.0350118 (PMC13322544; doi:10.1371/journal.pone.0350118)

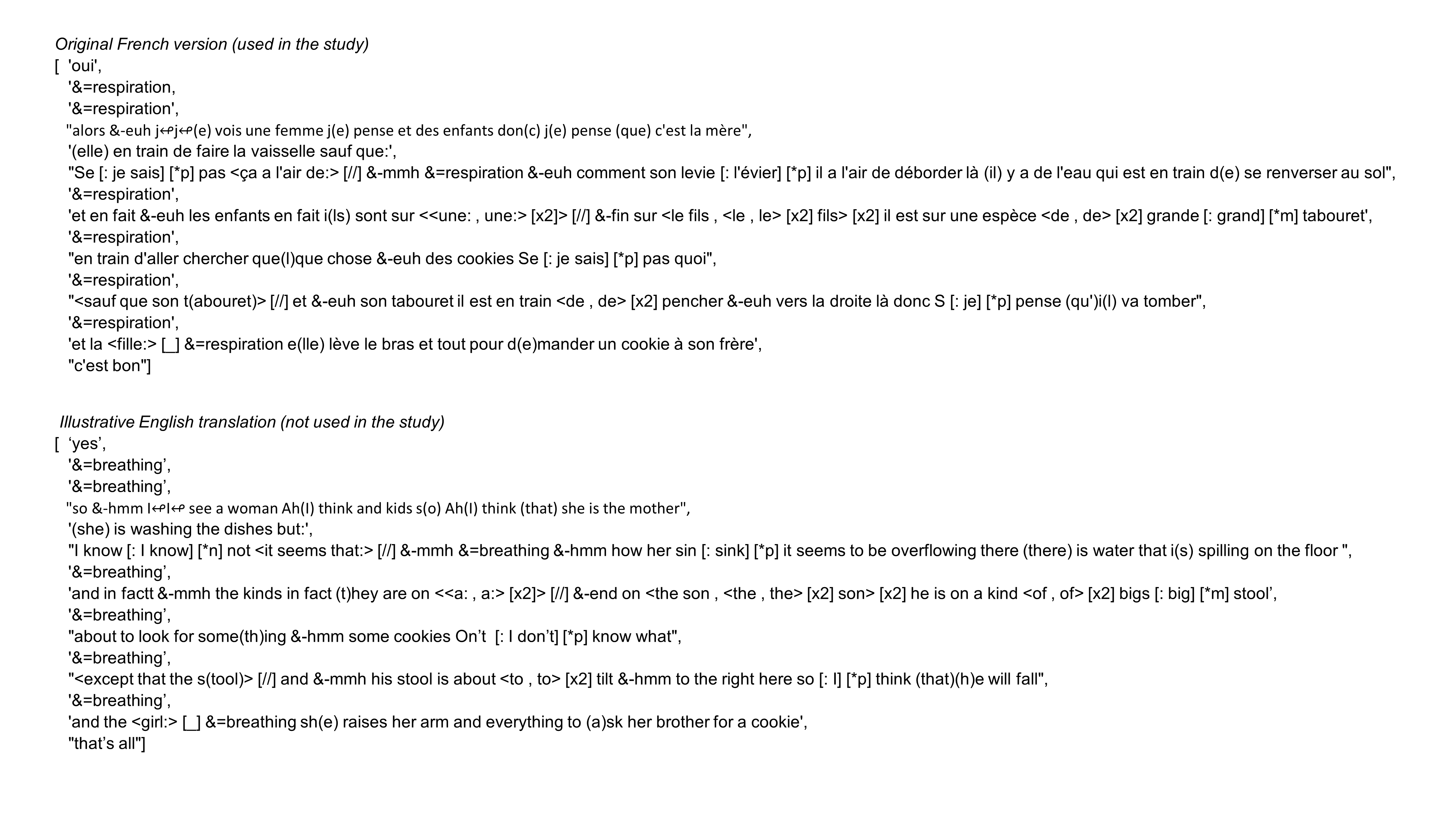

Supplement: S1 Fig — The second part is a translation in English that tried to keep the French errors. non intended productions (order of first apparition in the French example): ‘&= ‘: non linguistic additions, ‘&-’: filler, ‘↫’: stuttered word, ‘( )’: omitted word, ‘ [:] [*p]’: phonological distortion, ‘<> [//]’ revision (i.e., when participant change (revise) the intended sentence), ‘<> [x2]”: repetition, ‘ [:] [*p]’: morphological error, ‘ [_]’: abnormal prosody. (TIF) [file pone.0350118.s001.tif]

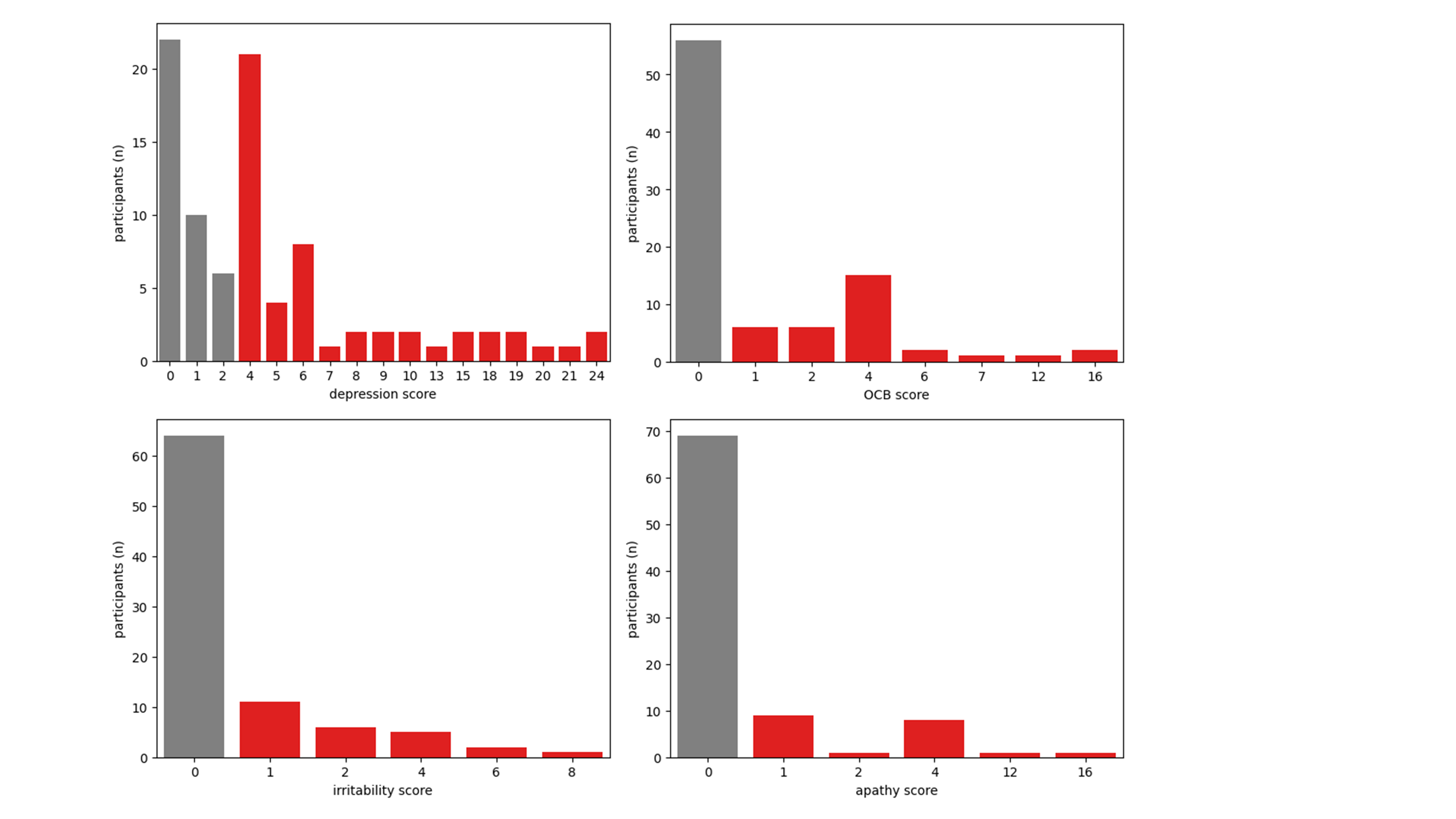

Supplement: S2 Fig — Grey bars indicate participants with PBA‑s scores below the median (classified as negative for the symptom), while red bars indicate participants with PBA‑s scores above the median (classified as positive for the symptom). (TIF) [file pone.0350118.s003.tif]

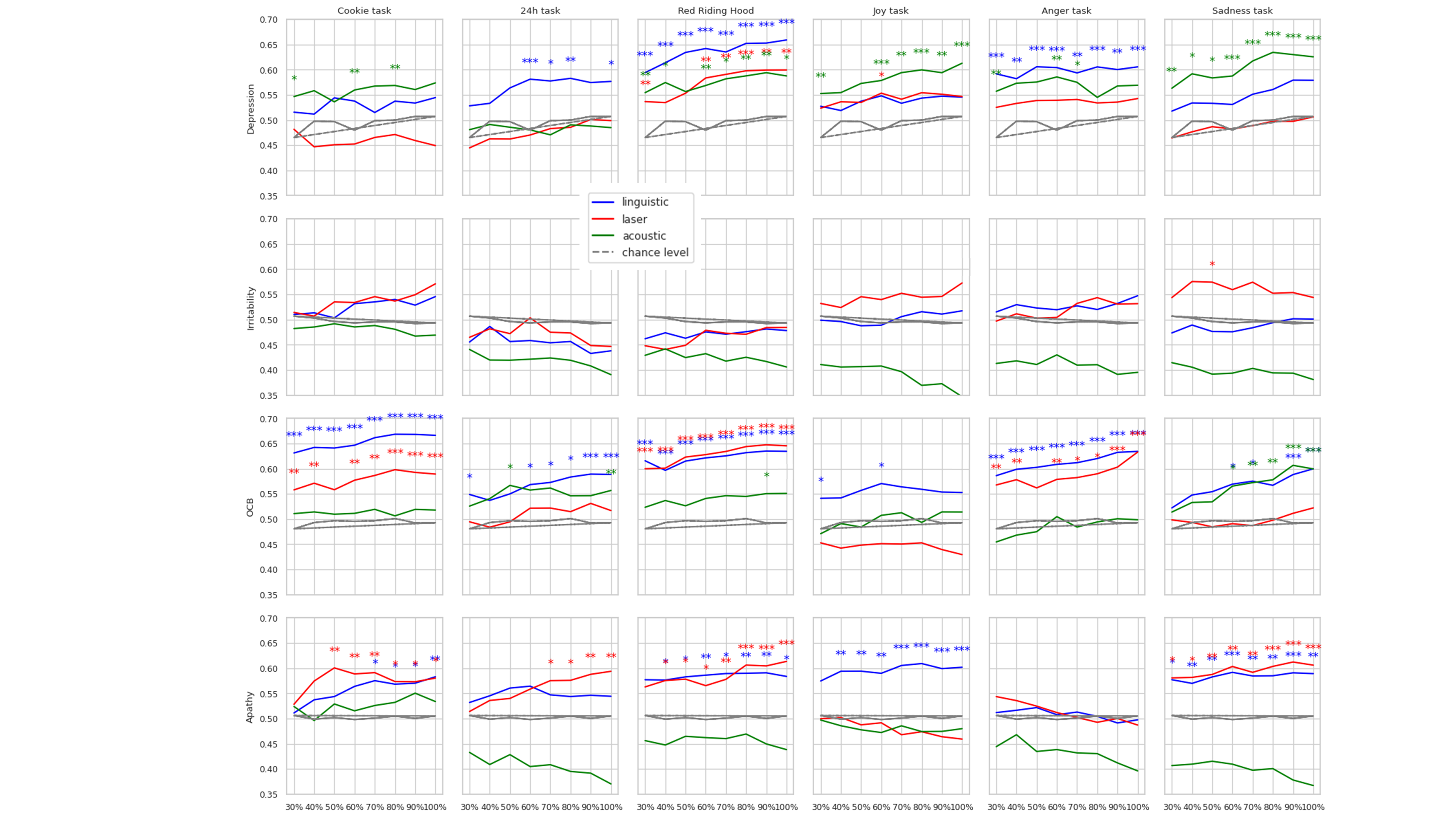

Supplement: S3 Fig — *: 0.01 < p ≤ 0.05, **: 0.001 < p ≤ 0.01, ***: p ≤ 0.001, these statistics were calculated only with the complete training set (same statistic than Fig 2 and Table 2) and are Bonferroni corrected for multiple comparison. (TIF) [file pone.0350118.s004.tif]

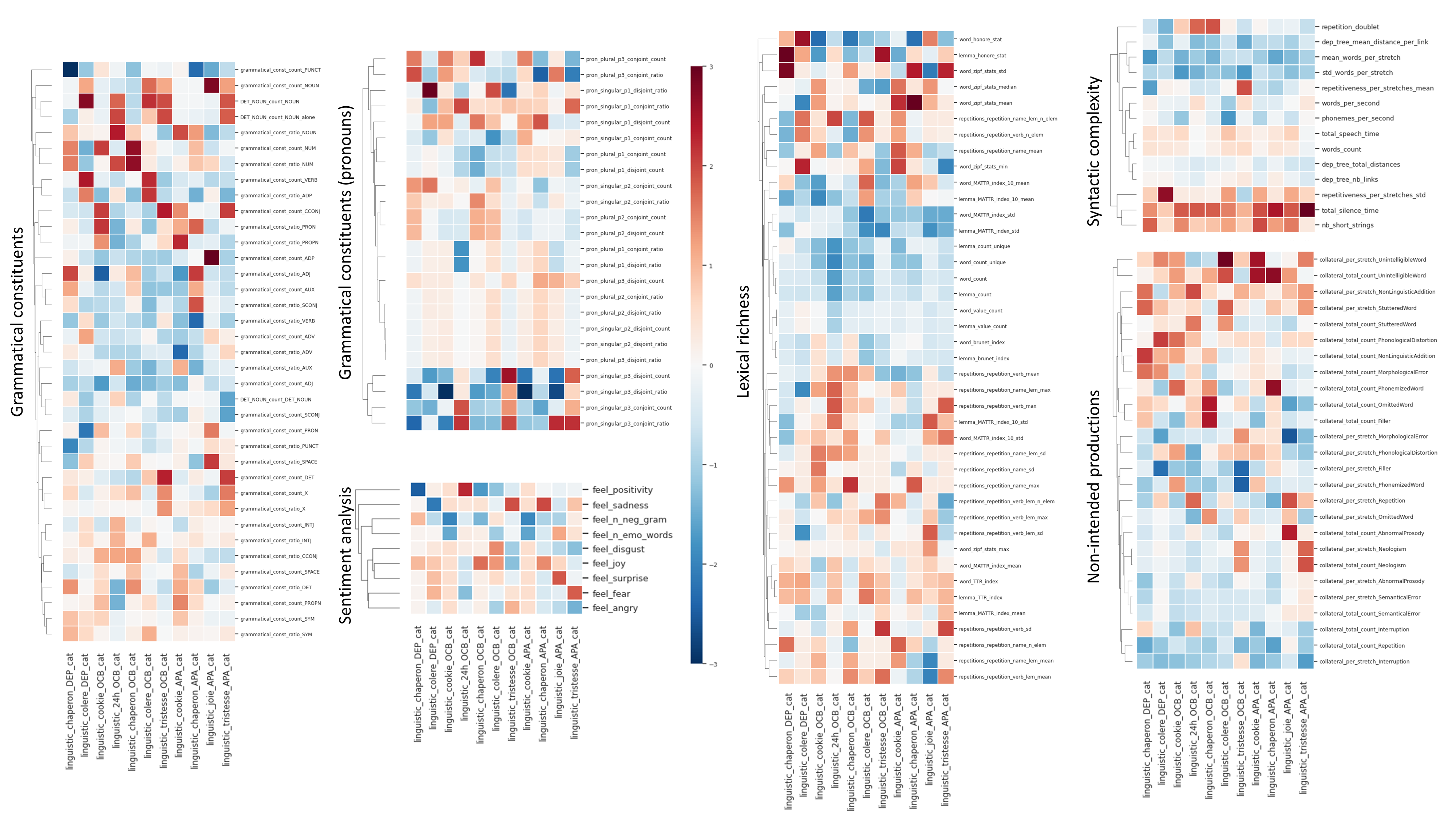

Supplement: S4 Fig — Logistic regression coefficients were converted to odds ratios and subsequently clustered within the sub‑categories of the linguistic feature sets to facilitate readability. Abbreviations: DEP depression, OCB obsessive compulsive behavior, APA apathy. (TIF) [file pone.0350118.s005.tif]

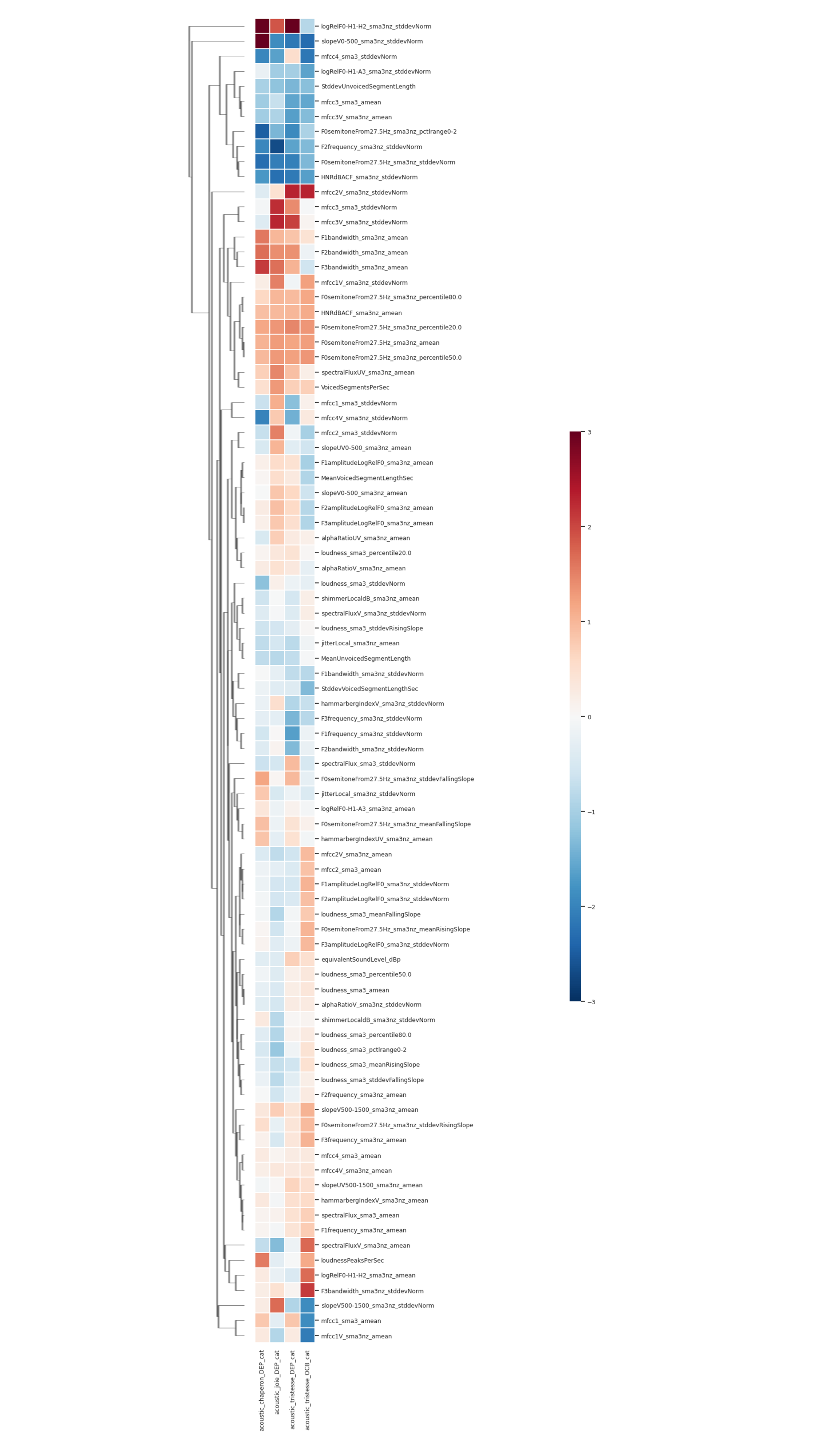

Supplement: S5 Fig — Logistic regression coefficients were converted to odds ratios and subsequently clustered to facilitate readability. Abbreviations: DEP depression, OCB obsessive compulsive behavior. (TIF) [file pone.0350118.s006.tif]
