# Supplementary material for: Development and validation of a machine learning model to detect psychiatric symptoms in Huntington’s disease using speech analysis
Source: PLoS One. 2026 Jul 1;21(7):e0350118. doi: 10.1371/journal.pone.0350118 (PMC13322544; doi:10.1371/journal.pone.0350118)
Supplement: S1 Table — The last row was Chance level. Abbreviations: DEP depression, IRR irritability, OCB obsessive compulsive behavior, APA apathy, se sensibility, sp specificity, ppv positive predictive value, npv negative predictive value, p_val p_value, NS non-significant. (DOCX) [file pone.0350118.s002.docx]

|  |  | Depression | | | | | | | Irritability | | | | | | | Obsessive/compulsive behavior | | | | | | | Apathy | | | | | | |
| --- | --- | --- | --- | --- | --- | --- | --- | --- | --- | --- | --- | --- | --- | --- | --- | --- | --- | --- | --- | --- | --- | --- | --- | --- | --- | --- | --- | --- | --- |
|  |  | F1-score | accuracy | se | sp | ppv | npv | p_val | F1-score | accuracy | se | sp | ppv | npv | p_val | F1-score | accuracy | se | sp | ppv | npv | p_val | F1-score | accuracy | se | sp | ppv | npv | p_val |
| **Cookie** | Linguistic f. | 0.54 | 0.56 | 0.63 | 0.47 | 0.60 | 0.51 | NS | 0.55 | 0.59 | 0.51 | 0.62 | 0.35 | 0.77 | NS | 0.67 | 0.68 | 0.67 | 0.68 | 0.58 | 0.77 | *** | 0.58 | 0.63 | 0.68 | 0.61 | 0.35 | 0.87 | * |
|  | Acoustic f. | 0.57 | 0.58 | 0.59 | 0.57 | 0.64 | 0.53 | NS | 0.47 | 0.50 | 0.52 | 0.49 | 0.28 | 0.72 | NS | 0.52 | 0.53 | 0.56 | 0.51 | 0.43 | 0.64 | NS | 0.53 | 0.62 | 0.47 | 0.67 | 0.29 | 0.82 | NS |
|  | Laser f. | 0.45 | 0.47 | 0.57 | 0.36 | 0.53 | 0.38 | NS | 0.57 | 0.65 | 0.41 | 0.75 | 0.40 | 0.76 | NS | 0.59 | 0.62 | 0.48 | 0.71 | 0.53 | 0.68 | *** | 0.58 | 0.70 | 0.39 | 0.79 | 0.37 | 0.82 | * |
| **24h** | Linguistic f. | 0.58 | 0.59 | 0.64 | 0.53 | 0.63 | 0.54 | NS | 0.44 | 0.50 | 0.36 | 0.55 | 0.22 | 0.69 | NS | 0.59 | 0.60 | 0.66 | 0.55 | 0.49 | 0.73 | ** | 0.54 | 0.60 | 0.62 | 0.59 | 0.31 | 0.85 | NS |
|  | Acoustic f. | 0.49 | 0.50 | 0.53 | 0.46 | 0.55 | 0.43 | NS | 0.39 | 0.42 | 0.40 | 0.43 | 0.21 | 0.64 | NS | 0.56 | 0.57 | 0.57 | 0.57 | 0.46 | 0.68 | NS | 0.37 | 0.45 | 0.26 | 0.50 | 0.12 | 0.70 | NS |
|  | Laser f. | 0.50 | 0.51 | 0.52 | 0.49 | 0.56 | 0.46 | NS | 0.45 | 0.55 | 0.23 | 0.67 | 0.22 | 0.69 | NS | 0.52 | 0.56 | 0.37 | 0.68 | 0.41 | 0.63 | NS | 0.59 | 0.72 | 0.39 | 0.82 | 0.38 | 0.82 | ** |
| **Red-riding hood** | Linguistic f. | 0.66 | 0.66 | 0.65 | 0.68 | 0.73 | 0.62 | *** | 0.48 | 0.53 | 0.42 | 0.57 | 0.27 | 0.72 | NS | 0.63 | 0.64 | 0.69 | 0.61 | 0.54 | 0.76 | *** | 0.58 | 0.65 | 0.62 | 0.65 | 0.35 | 0.86 | * |
|  | Acoustic f. | 0.59 | 0.61 | 0.68 | 0.51 | 0.65 | 0.56 | * | 0.41 | 0.44 | 0.39 | 0.46 | 0.22 | 0.66 | NS | 0.55 | 0.56 | 0.64 | 0.51 | 0.45 | 0.70 | NS | 0.44 | 0.50 | 0.40 | 0.53 | 0.20 | 0.75 | NS |
|  | Laser f. | 0.60 | 0.61 | 0.66 | 0.55 | 0.65 | 0.57 | ** | 0.48 | 0.58 | 0.28 | 0.70 | 0.27 | 0.72 | NS | 0.65 | 0.67 | 0.58 | 0.72 | 0.60 | 0.74 | *** | 0.61 | 0.73 | 0.46 | 0.80 | 0.41 | 0.84 | *** |
| **Joy** | Linguistic f. | 0.55 | 0.56 | 0.61 | 0.50 | 0.61 | 0.50 | NS | 0.52 | 0.56 | 0.53 | 0.56 | 0.32 | 0.76 | NS | 0.55 | 0.57 | 0.55 | 0.58 | 0.46 | 0.67 | NS | 0.60 | 0.65 | 0.70 | 0.63 | 0.37 | 0.89 | *** |
|  | Acoustic f. | 0.61 | 0.62 | 0.63 | 0.60 | 0.68 | 0.58 | *** | 0.35 | 0.37 | 0.39 | 0.37 | 0.18 | 0.61 | NS | 0.51 | 0.53 | 0.48 | 0.57 | 0.41 | 0.64 | NS | 0.48 | 0.55 | 0.42 | 0.59 | 0.23 | 0.79 | NS |
|  | Laser f. | 0.55 | 0.56 | 0.62 | 0.48 | 0.60 | 0.51 | NS | 0.57 | 0.66 | 0.42 | 0.75 | 0.39 | 0.77 | NS | 0.43 | 0.48 | 0.27 | 0.61 | 0.31 | 0.56 | NS | 0.46 | 0.65 | 0.15 | 0.79 | 0.16 | 0.76 | NS |
| **Anger** | Linguistic f. | 0.61 | 0.61 | 0.60 | 0.63 | 0.67 | 0.56 | ** | 0.55 | 0.59 | 0.54 | 0.61 | 0.35 | 0.78 | NS | 0.63 | 0.64 | 0.70 | 0.61 | 0.54 | 0.76 | *** | 0.50 | 0.54 | 0.59 | 0.53 | 0.26 | 0.82 | NS |
|  | Acoustic f. | 0.57 | 0.58 | 0.55 | 0.60 | 0.64 | 0.52 | NS | 0.40 | 0.43 | 0.38 | 0.45 | 0.20 | 0.66 | NS | 0.50 | 0.52 | 0.45 | 0.56 | 0.39 | 0.62 | NS | 0.40 | 0.50 | 0.22 | 0.58 | 0.13 | 0.72 | NS |
|  | Laser f. | 0.54 | 0.55 | 0.56 | 0.54 | 0.60 | 0.50 | NS | 0.53 | 0.62 | 0.36 | 0.73 | 0.32 | 0.75 | NS | 0.63 | 0.68 | 0.46 | 0.81 | 0.63 | 0.71 | *** | 0.49 | 0.69 | 0.17 | 0.83 | 0.21 | 0.78 | NS |
| **Sadness** | Linguistic f. | 0.58 | 0.59 | 0.61 | 0.56 | 0.64 | 0.54 | NS | 0.50 | 0.53 | 0.54 | 0.53 | 0.31 | 0.75 | NS | 0.60 | 0.61 | 0.65 | 0.58 | 0.50 | 0.73 | ** | 0.59 | 0.63 | 0.73 | 0.60 | 0.35 | 0.89 | ** |
|  | Acoustic f. | 0.63 | 0.64 | 0.67 | 0.59 | 0.68 | 0.60 | *** | 0.38 | 0.40 | 0.42 | 0.40 | 0.21 | 0.64 | NS | 0.60 | 0.61 | 0.63 | 0.60 | 0.51 | 0.72 | *** | 0.37 | 0.44 | 0.24 | 0.50 | 0.12 | 0.69 | NS |
|  | Laser f. | 0.51 | 0.52 | 0.58 | 0.44 | 0.56 | 0.46 | NS | 0.54 | 0.64 | 0.35 | 0.75 | 0.36 | 0.75 | NS | 0.52 | 0.57 | 0.38 | 0.68 | 0.44 | 0.63 | NS | 0.61 | 0.76 | 0.34 | 0.88 | 0.48 | 0.82 | *** |
| **Chance** |  | 0.51 | 0.51 | 0.56 | 0.45 | 0.56 | 0.45 | NS | 0.49 | 0.59 | 0.27 | 0.72 | 0.27 | 0.72 | NS | 0.49 | 0.52 | 0.38 | 0.61 | 0.38 | 0.61 | NS | 0.51 | 0.63 | 0.29 | 0.73 | 0.23 | 0.78 | NS |
