# Supplementary material for: Development and validation of a machine learning model to detect psychiatric symptoms in Huntington’s disease using speech analysis
Source: PLoS One. 2026 Jul 1;21(7):e0350118. doi: 10.1371/journal.pone.0350118 (PMC13322544; doi:10.1371/journal.pone.0350118)
Supplement: S1 File — (DOCX) [file pone.0350118.s007.docx]

**Linguistic Features**

The pipeline extracted a set of 151 linguistic features with **structural, lexical, syntactic, emotional, and collateral features** from speech identified from literature [8–13,21]. These features were extracted from the annotated string text of speech content. It goes beyond simple word counts by quantifying **grammatical composition, syntactic distances, lexical diversity indices, repetition patterns, emotional valence, and anomalies in production**. They were quantified by their absolute number of occurrences on the whole task (and when specified with a mean, standard deviation, minimum or maximum value by task or stretch). This comprehensive set of features provides a rich linguistic fingerprint of each participant’s narrative speech and was split into five groups:

**1. Grammatical Constituents**

- **PoS counts and ratios**: Major Part of speech (PoS) categories (NOUN, VERB, PRON, ADV, DET, CCONJ, SCONJ, AUX, ADP, ADJ) were automatically identified using spaCy's ('fr core news lg') [34]. Frequency and relative proportions were calculated (grammatical_const_count, grammatical_const_ratio).
- **Nouns with determiners**: Distinction between bare nouns and nouns preceded by determiners (DET_NOUN_count).
- **Pronoun morphology:** Counts and ratios of pronouns classified by person, number, and morphological attributes (pers_pron_count, pers_pron_ratio) such as as first, second or third person, singular or plural, disjunctive.

**2. Syntactic Complexity**

- **Words count:** Total number of words produced in a task (words_count).
- **Words per second**: Ratio of words to total speech duration (words_per_second).
- **Phonemes per second**: Ratio of phonemes to duration (phonemes_per_second).
- **Short stretches**: Number of utterances containing <3 words (nb_short_strings).
- **Speech vs. silence time**: Total speech duration vs. silence duration (total_speech_time, total_silence_time).
- **Dependency tree metrics** [35]: calculated using spaCy
  - Total syntactic link distances (dep_tree_total_distances).
  - Number of syntactic links (dep_tree_nb_links).
  - Mean dependency distance per link (dep_tree_mean_distance_per_link).
- **Repetitiveness across stretches**: using cosine similarity between spaCy embeddings of stretches, yielding mean and standard deviation (repetitiveness_per_stretches_mean, repetitiveness_per_stretches_std).
- **Repetition doublets**: Specific detection of repetitions occurring exactly twice (repetition_doublet).

**3. Lexical Richness**

- **Unique words per stretch**: Mean and standard deviation of unique word counts (word_count_unique, lema_count_unique), with both on lexeme (surface) forms and lemmas
- **Word frequency statistics**: using wordfreq[36], Zipf scale values (median, mean, min, max, std) (word_zipf_stats) were calculated.
- **Lexical repetition**: Counts of nouns and verbs repeated more than once, computed both on lexemes and lemmas (repetitions). Mean, standard deviation, maximum repetition count, and number of repeated elements were calculated.
- **Lexical diversity indices** [11]: with both on lexemes and lemmas
  - **Type–Token Ratio (TTR)** (word_TTR_index, lemma_TTR_index).
  - **Moving‑Average TTR (MATTR)** with window sizes of 50 and 10 words, reporting mean and std (word_MATTR_index_mean/std, word_MATTR_index_10_mean/std).
  - **Honore’s Statistic** (word_honore_stat, lemma_honore_stat).
  - **Brunet’s Index** (word_brunet_index, lemma_brunet_index).

**4. Sentiment analysis**

- **Emotion lexicon analysis (FEEL)**: Aggregated values for positivity, joy, fear, sadness, anger, surprise, and disgust (feel) using a dedicated French lexicon[37].
- **Negation markers**: Counts of grammatical negations (e.g., *ne…pas*) (n_neg_gram).

**5. Non‑Intended Productions (Collateral Track)**

- **Annotations of anomalies**: Counts of fillers, pauses, interruptions, phonological distortions, semantic errors, morphological errors, neologisms, repetitions, abnormal prosody, omitted words, stuttered words, phonemized words, unintelligible words, and non‑linguistic additions (collateral_total_count, collateral_per_stretch). All these productions were annotated by speech therapists. S1 Fig contains examples of such non intended productions.
- **Aggregate measures**: Number of stretches containing each anomaly, total counts across the task.

## LASER Feature

We used **LASER (Language‑Agnostic SEntence Representations)** [31], a multilingual sentence embedding model where semantically similar utterances are represented closely in a Euclidean space (vector space of 1024 dimensions), regardless of language. We applied the French encoder (fra_Latn) to all stretches of annotated string text of speech content. This allows the model to exploit semantic patterns in narrative tasks, potentially revealing psychiatric symptomatology through differences in content coherence, variability, or thematic focus.

### 1. ****Input Processing****

- **Patient ‘intended form’**: Each narrative stretch was transcribed by speech therapists and cleaned to remove empty strings or null values.
- **Encoding**: Each stretch was passed through LASER to obtain a 1024‑dimensional embedding vector.

### 2. ****Feature Representations****

We derived three types of LASER features:

- **Mean embedding**
  - Computed as the mean across all stretches of the 1024-dimensional vectors.
  - Would capture a mean semantic profile between stretches.
- **Standard deviation embedding**
  - Computed as the standard deviation across all stretches of the 1024-dimensional vectors.
  - Would reflect variability in semantic content between stretches.
- **Concatenated embedding**
  - All stretches concatenated into a single long text string, then encoded into a single 1024‑dimensional vector.
  - Would provide a global representation of the entire narrative task, independent of stretch segmentation.

This resulted to three sets of 1024 features, thus 3072 LASER features (mean, SD, and concatenated).

## Acoustic Feature

We extracted acoustic features using the **openSMILE toolkit** (version eGeMAPSv02, Functionals level) [38], a standardized set of parameters designed for emotion and mental state recognition in speech. This set includes 88 low level descriptors (LLDs) and their statistical functionals to capture prosody, voice quality, spectral composition, and temporal dynamics of speech. It was developed as an international consensus baseline for affective computing and clinical speech analysis. These acoustic descriptors were created by combining all task segments into a single continuous audio signal rather than using the entire original audio file in order to prevent any possible non-participant noises.

### 1. ****Low‑Level Descriptors (LLDs)****

The following categories of acoustic parameters are extracted from the raw audio signal:

- **Prosodic features**
  - Fundamental frequency (F0, pitch) mean, standard deviation, percentiles, range.
  - Loudness (energy) mean, std, percentiles.
  - Relative loudness peaks and slopes.
- **Voice quality features**
  - Jitter (cycle‑to‑cycle variation in F0).
  - Shimmer (cycle‑to‑cycle variation in amplitude).
  - Harmonics‑to‑Noise Ratio (HNR).
  - Alpha ratio, Hammarberg index, spectral slope.
- **Spectral features**
  - Formant frequencies and bandwidths (F1, F2, F3).
  - Spectral flux, centroid, entropy.
  - MFCCs (Mel‑Frequency Cepstral Coefficients) 1–4.
- **Temporal features**
  - Speech rate proxies (syllable rate, voiced/unvoiced segment durations).
  - Voicing probability statistics.
  - Length and distribution of pauses.

### 2. ****Functionals****

For each LLD, statistical functionals are computed over the entire segment or concatenated task:

- Mean, standard deviation, variance.
- Percentiles (25th, 50th, 75th).
- Range (max–min).
- Linear regression coefficients (slope, offset).
- Position of maxima/minima.
